# Comparison of Peripheral and Central Schizophrenia Biomarker Profiles

**DOI:** 10.1371/journal.pone.0046368

**Published:** 2012-10-30

**Authors:** Laura W. Harris, Sandra Pietsch, Tammy M. K. Cheng, Emanuel Schwarz, Paul C. Guest, Sabine Bahn

**Affiliations:** 1 Department of Chemical Engineering and Biotechnology, University of Cambridge, Cambridge, United Kingdom; 2 Department of Neuroscience, Erasmus MC, Rotterdam, The Netherlands; Chiba University Center for Forensic Mental Health, Japan

## Abstract

We have recently shown that a molecular biomarker signature comprised of inflammatory, hormonal and growth factors occurs in the blood serum from first onset schizophrenia patients. Here, we use the same platform to investigate post mortem brain tissue (Brodmann area 10) from schizophrenia patients who were mainly chronically ill and drug treated. Twenty-one analytes are differentially expressed in post-mortem brain tissue. Comparison with our previous mRNA profiling studies of the same patient samples in another frontal cortical area showed that 9 of these molecules were also altered at the transcriptional level. Furthermore, 9 of the molecules were also altered in serum from living first onset schizophrenia patients compared to controls. We propose a model in which the brain and periphery are coordinated through hormones and other regulatory molecules released into the blood via the diffuse neuroendocrine system. These findings provide further evidence for the systemic nature of schizophrenia and give added validity to the concept that schizophrenia can be investigated through studies of blood-based biomarkers.

## Introduction

Schizophrenia is widely considered to be a heterogeneous disorder with multiple causes. Diagnosis is made by means of interview and despite some advances, no biological features with clear diagnostic or prognostic power are known. This hinders accurate diagnosis and thereby precludes the known benefits of early treatment [1], [2]. Although primarily considered a disorder of the central nervous system, a peripheral component has been demonstrated for schizophrenia [3], [4], [5], [6], [7]. However the relationship between central and peripheral features is not understood.

Recently, we described the identification of a panel of blood-based biomarkers which has high specificity and sensitivity in distinguishing schizophrenia patients from controls, using a multiplex immunoassay approach [3], [8]. This panel consists of proteins and small molecules related to metabolism, endocrine function and inflammation, many of which have not been described previously in schizophrenia. It is therefore of interest to conduct a parallel investigation in brain tissue from schizophrenia patients, to identify which, if any, of these markers are differentially expressed in brain tissue. Furthermore, since circulating factors such as hormones influence brain function, it is of interest to identify which of these molecules link central and peripheral function in schizophrenia.

Here, we use the same multiplex immunoassay platform to investigate *post mortem* brain tissue from schizophrenia patients in comparison to molecules identified in the serum of first onset schizophrenia patients and those reported in the literature. The aims of this study are to investigate the relationship between brain and periphery in schizophrenia and the potential role of the serum markers in schizophrenia symptomatology, and to add their validity as diagnostic markers.

## Materials and Methods

### Biochemicals and reagents

All biochemicals and reagents were obtained from Sigma-Aldrich (Poole, Dorsett, UK) unless stated otherwise.

### Ethics statement

Brain tissue was obtained from the Stanley Medical Research Institute. Tissue was collected *post mortem* from patients and controls with full informed consent obtained from a first degree relative after death in compliance with the Declaration of Helsinki. In each case consent was obtained by questionnaires conducted over the phone (since the next-of-kin were not on the premises of the Medical Examiners Office at the time of autopsy) and signed by two witnesses. The protocol was reviewed and accepted by the Uniformed Services University of Health Sciences institutional review board (IRB) and exemption from formal approval was granted on the grounds that specimens were obtained via informed donation from cadaveric material in accordance with federal and state regulations, the research did not encompass genetic linkage studies, and all samples were de-identified and personal information anonymised. Local ethical approval for use of this tissue was granted by the Cambridgeshire 2 Local Research Ethics Committee.

### Brain tissue

Fresh-frozen brain tissue blocks (Brodmann area 10) of schizophrenia, bipolar disorder, and psychiatrically normal control individuals were obtained from the Stanley Medical Research Institute Array Collection (Table 1). Sample identities were coded, blinded and randomised with respect to diagnosis to minimize experimental or analytical bias. For continuous demographic variables (age, pH, PMI) group differences between schizophrenia and control samples were assessed by t-test. For gender, group differences were assessed by Fisher's exact test. Approximately 100 mg tissue from each block was minced into 10–20 ug fragments on dry ice and combined with freshly prepared lysis buffer [50 mM Tris- HCl (pH 7.4), containing Complete Mini Protease Inhibitor Cocktail Tablets and Pepstatin (Roche Diagnostics Ltd, UK) in pre- chilled Lysing Matrix Tubes, type D (MP Biomedicals; Cedex, France) at a ratio of 1 g tissue/9 mL buffer. The tissues were homogenized twice for 20 seconds using a FastPrep®-24 Instrument (MP Biomedicals) at a setting of 4.5 and placed on ice for 30 seconds. The homogenates were transferred to Eppendorf LoBind Protein tubes (Fisher Scientific Ltd; Loughborough, UK) and centrifuged at 4°C for 3 minutes at 10,000× G in a refrigerated Heraeus Fresco17 Centrifuge (Fisher Scientific Ltd). The supernatants were collected and stored at −80°C. Protein concentrations were determined using a DC Protein Assay according to manufacturer's instructions (Bio-Rad; Hemel Hempstead, UK).

**Table 1:** Demographic details of brain tissue samples.

	Control (n = 33)	Schizophrenia (n = 35)	Bipolar disorder (n = 33)
**Gender (M/F)**	25/8	26/9	15/18*
**Age (years)**	44.8±7.3	42.6±8.5	45.4±10.8
**Brain pH**	6.61±0.27	6.47±0.24*	6.44±0.30*
**Post mortem interval (hours)**	29.6±13.2	31.4±15.5	38.0±18.9*
**Lifetime antipsychotics (fluphenazine mg equivalent)**	0	85004±100335	9915±23158
**Duration of illness (years)**	na	21±10.1	20±9.6

In schizophrenia samples, no significant differences were observed in age (p = 0.241), gender (p = 0.990) or PMI (0.610), however there was a significant difference in brain pH (p = 0.028). In bipolar disorder samples there were no significant difference in age (p = 0.801) but brain pH, PMI and gender differed significantly from control (p = 0.015, 0.044 and p = 0.023 respectively).

### Multiplex immunoassay analysis

Extracts (200 µL) were analysed using the DiscoveryMAP™ multiplexed immunoassay panel at Myriad-RBM (Austin, TX, USA), comprising assays for 187 molecules (Table S1). Each assay was calibrated using duplicate 8-point standard curves, and raw intensity measurements were interpreted into final protein concentrations using proprietary software. Machine performance was verified using quality control samples at low, medium, and high levels for each analyte.

### Statistical analysis

Principal component analysis (PCA) (Umetrics AB; SIMCA-P+ 12.0) was used to identify outliers. This showed that 3 samples lay outside the confidence ellipse and were removed from further analysis. Values which were outside the detection limits were replaced with the minimum or maximum value for each assay as appropriate. Assays showing less than 33% detectable values were excluded from the analysis, leaving a total of 172 analytes available for analysis. Further statistical analysis was carried out using SPSS Statistics 17.0 (IBM). Correlation analysis was carried out to investigate the effect of the demographic variables on the data. Significant associations between the assay results, pH, PMI, age and gender were identified and these variables were incorporated into an ANCOVA model. The false discovery rate (FDR) was controlled using the method of Benjamini and Hochberg [9]. Adjusted p values are denoted as q values throughout. Following adjustment by ANCOVA, fold change was derived from the estimated marginal mean of each group and expressed as mean/control.

### Haemoglobin immunoassay

A Quantichrom™ Hemoglobin Assay Kit (BioAssay Systems; Hayward, California, USA) was used to determine haemoglobin levels in the supernatants as an indicator of the levels of blood contamination in the brain samples. The assay was performed on clear-bottom 96-well plates according to the manufacturer's recommendations. Briefly, 50 µL aliquots of each sample were diluted at a ratio of 1∶2 with 50 mM Tris-HCl buffer, combined with 200 µL kit reagent and incubated for 5 minutes at room temperature. Optical densities were determined at 400 nm using a μQuant™ spectrophotometer (BioTek; Potton, UK). These readings were converted to concentrations by comparison the readings of a 5–75 mg/dL Hemoglobin Calibrator standard curve. All samples were assayed in duplicate.

### Uniplex immunoassays

Selected hits from the MAP analysis were replicated in-house using individual enzyme-linked immunoadsorbent assays (ELISAs). These were performed on a subset of samples chosen according to their availability. At least 15 samples per group of schizophrenia and control samples were extracted using the method described above. ELISAs were performed for interferon gamma (Abcam; Cambridge, UK), chromogranin A (ALPCO Diagnostics; Salem, NH, USA) and tissue inhibitor of metalloproteinases-1 (TIMP-1) (Abnova; Tapei, Taiwan) according to the manufacturers' instructions.

### Comparison with transcriptome data

MAP data was compared to microarray data derived from brain tissue (BA9; [10]) and cerebral microvascular endothelial cells [11] from the same subjects. Full details of the microarray experiments can be found in the relevant manuscripts. MAP analyte names were converted to SwissProt identifiers and used to query the Affymetrix database (NetAffx; www.affymetrix.com). As cortisol was one of the most significant analytes detected using the MAP analyses, we also determined the expression level of mRNA encoding the glucocorticoid receptor NR3C1. For correlation analysis, raw MAP data were first log2 transformed for comparability to the normalised microarray data. MAP data and microarray transcriptomic data for the same subjects were then tested using Pearson's correlation within each diagnostic group.

### Comparison with serum data

Data derived from brain tissue samples were compared to those from our previously published analysis of serum samples from 250 recent-onset schizophrenia patients, and 230 control subjects [3] (Table 2). For this dataset [3], ethical committees from the Universities of Cologne (cohort 1), Muenster (cohort 2) and Magdeburg (cohorts 3 and 5) in Germany, and that from Erasmus University (cohort 4) in the Netherlands, approved the protocols of the study. Informed written consent was obtained for all participants, diagnoses were carried out using the Diagnosis and Statistical Manual (DSM)-IV and clinical tests performed by psychiatrists. All patients selected for the study had the paranoid subtype of schizophrenia (DSM-IV 295.30) with no other medical conditions. Controls were recruited from geographical areas matching the patient populations and those with no family history of mental disease or other medical conditions were selected for the study. Briefly, non-parametric, two-tailed Wilcoxon rank-sum tests were carried out to identify significant expression differences between patients and controls as described in Schwarz et al [3]. For the purposes of comparison with the brain dataset, we wished to use the most reproducible set of markers, therefore we only considered molecules which were replicated in at least 2 cohorts.

**Table 2:** Demographic details for the serum cohorts [3].

*Cohort no:*	SCZ1	SCZ2	SCZ3	SCZ4	SCZ5	BPD1
**Patients n**	71	46	46	47	40	32
**Controls n**	59	46	45	40	40	59
**Patients (M/F)**	42/29	35/11	30/16	36/11	27/12	13/19
**Controls (M/F)**	31/28	35/11	27/18	33/7	26/14	31/28
**Patients Age**	31±10	27±9	35±12	26±8	35±10	34±10
**Controls Age**	30±8	27±9	34±12	27±4	36±11	30±8
**Patients BMI**	24±5	22±2	26±5	na	25±5	25±4
**Controls BMI**	23±4	na	24±4	na	24±3	23±4
**Patients Smoking (Y/N/na)**	25/23/23	16/26/4	25/21/0	33/14/0	22/18/0	7/14/11
**Controls Smoking (Y/N/na)**	25/34/0	na	11/34/0	na	18/22/0	25/34/0
**Patients Cannabis (Y/N/na)**	33/22/16	15/27/4	8/38/0	23/24/0	7/33/0	7/14/11
**Controls Cannabis (Y/N/na)**	31/25/3	na	0/45/0	na	3/37/0	31/25/3
**Medication free patients**	all	all	33	all	all	4
**PANSS positive item score**	23±6	18±7	21±5	na	23±7	na
**PANSS negative item score**	23±8	18±7	22±7	na	19±8	na

Schizophrenia subjects are first or recent-onset as described in [3]. The same controls are used for SCZ1 and BPD1. Values are shown as mean±s.d. Abbreviations: BMI, body mass index; M/F, male/female; na, not available; PANSS, positive and negative syndrome scale; SCZ, schizophrenia; Y/N, yes/no.

### Targeted analyte clustering

A targeted analyte cluster (TAC) approach was applied to identify molecules which show correlated expression levels in the datasets, as described previously (Cheng *et al*, 2010). In this method, correlated analytes are identified by analyzing the data in reproducing kernel spaces. Analytes were pre-classified into broad categories of “metabolic”, “immune” and “other” analytes, based on functional annotation database searching in order to reduce the datasets to a manageable size as well as to increase the likelihood of identifying biologically relevant clusters. The analysis was then performed within these categories, plus “metabolic” and “immune” analytes together and all analytes together. TAC examined small analyte clusters within each category and ranked their precision (true positive predictions divided by the sum of true positive and false positive predictions) in terms of distinguishing schizophrenia from control subjects in brain tissue. We then tested the top clusters in the serum data (cohort 1 only), in order to identify clusters which had similar discriminatory power in serum as in brain. The analysis was then performed in reverse, by identifying correlated analyte clusters in the serum data which were then tested on the brain dataset to determine if these could separate schizophrenia and control subjects. To further identify clusters that specifically give a good prediction power in distinguishing schizophrenia from controls but not bipolar disorder from controls, we also tested the ability of those schizophrenia-associated clusters to separate bipolar disorder patients from controls. In addition, interactions between members of a cluster were investigated *in silico* using the Ingenuity Pathways Knowledgebase (www.ingenuity.com).

## Results

### Multiplex immunoassay analyses of brain tissue

Analysis of schizophrenia and control brain tissue using multiplexed immunoassay profiling identified 21 analytes which showed significant differences (p<0.05) after adjusting for covariates using ANCOVA (see Table S2 for details of the covariate analysis). The false discovery rate-adjusted q values ranged between 0.17 and 0.4. A potential confounding factor in this analysis lies in the range of analytes tested, which primarily target circulating factors. As the brain tissue was dissected post mortem, samples contain blood as well as tissue. It is expected that in the majority of cases any contaminating blood will have been washed out and the ratio of blood to tissue will be extremely small, meaning that measured analyte expression levels will be derived principally from the tissue. However if a high degree of variability exists in the amount of blood contamination between samples, an artefactual difference in analyte levels between samples may be detected. We used a haemoglobin assay as a proxy measure for the amount of blood contamination in each tissue sample. Samples contained 34.0+/−17.5 mg/dl Hb with no significant difference between schizophrenia and control samples (p = 0.43). However, 5 analytes showed significant correlations with haemoglobin levels. Incorporation of Hb levels into the ANCOVA model showed that only 3 of 21 analytes were no longer significant and 2 new analytes became significant. False discovery rate-adjusted q values ranged between 0.28 and 0.45 (Table 3). The largest fold changes were seen for C-reactive protein (8.3-fold), interferon-γ (2.5-fold), fibroblast growth factor-4 (−2.5-fold), macrophage inflammatory protein-1b (−2.8-fold) and glutathione S-transferase [−3.3-fold; which reached significance only after ANCOVA adjustment for Hb level (p = 0.0.050)]. These findings suggested alterations in inflammatory pathways in the brain which were not explained by differing levels of blood contamination.

**Table 3:** Identification of altered molecules in brain tissues from schizophrenia and control subjects using multiplex immunoassay analysis.

					*BA9 homogenate microarray data*		*BA9 endothelial microarray data*		*serum data (all centres)*	
*Analyte name*	*SwissProt/Pubchem ID*	*ANCOVA p value*	*FC (adj)*	*Affymetrix probe ID*	*p value*	*FC*	*p value*	*FC*	*no of centres significant*	*mean FC*
**C Reactive Protein**	P02741	**0.027**	**8.3**	205753_at	n/s		n/s		**3**	**3.1**
Interferon-γ	P01579	0.008	2.5	210354_at	n/s		n/s		1	1.6
**Cortisol**	5754	**0.003**	**1.8**	201865_x_at/211671_s_at	**0.006(NR3C)**	**−1.7**	**0.001 (NR3C)**	**11.6**	**5**	**1.3**
**Resistin**	Q9HD89	**0.043**	**1.8**	220570_at	n/s		n/s		**3**	**1.2**
Adiponectin*	Q15848	0.057	1.7	207175_at	n/s		n/s		0	
**VCAM-1**	P19320	**0.044**	**1.3**	203868_s_at	n/s		**0.045**	**−5.4**	0	
**TIMP-1**	P01033	**0.045**	**1.3**	201666_at	**0.014**	**2.0**	n/s		**3**	**1.1**
PARC (CCL18)*	P55774	0.069	1.3	209924_at	n/s		n/s		0	
**Prostatic Acid Phosphatase**	P15309	**0.049**	**1.1**	204393_s_at	n/s		**0.036**	**−1.4**	1	−1.3
**Cancer Antigen 125**	Q8WX17	**0.048**	**−1.1**	220196_at	n/s		**0.056**	**−1.4**	0	
**Chromogranin A**	P10645	**0.027**	**−1.1**	204697_s_at	**0.014**	**−1.48**	n/s		**3**	**1.5**
Human β (CC) chemokine-4	O15467	0.005	−1.1	207354_at	n/s		n/s		0	
**VEGF**	P15692	**0.024**	**−1.2**	210513_s_at	n/s		n/s		**3**	**1.1**
**Thrombopoietin**	P40225	**0.011**	**−1.2**	211154_at	**0.043**	**1.1**	n/s		**2**	**1.0** +
**Alpha-2 Macroglobulin** ∧	PO1023	**0.029**	**−1.3**	217757_at	n/s		n/s		**2**	**1.2**
**Immunoglobulin E** *	P01854	**0.053**	**−1.4**	1558438_a_at	not measured		**0.035**	**−1.7**	0	
Matrix metalloproteinase-1	P03956	0.003	−1.4	204475_at	n/s		n/s		not measured	
Cancer Antigen 19-9	Q9BXJ9	0.016	−2.2	not measured					1	1.4
Fibroblast growth factor-4	P08620	0.018	−2.5	206783_at	n/s		n/s		0	
**MIP-1β(CCL4)**	P13236	**0.035**	**−2.8**	204103_at	n/s		**0.052**	**−1.1**	1	−1.3
**Glutathione S-Transferase alpha** ∧	PO8263	**0.050**	**−3.2**	215766_at	n/s		n/s		**3**	**1.1**

Columns 3–4 show p values and fold change (FC) values between schizophrenia and control groups by ANCOVA, including age, brain pH, PMI and haemoglobin level as covariates.

* Indicates loss of significance after the adjustment for haemoglobin level,

∧ indicates significance achieved only after adjustment for haemoglobin level. The list of altered brain analytes was compared with published microarray mRNA transcript data (column 5 shows the Affymetrix probes used; for cortisol, the probe for the associated NR3C receptor was used; for genes with more than one probe, only the most significant probe is shown). Columns 6–9 show p values and fold changes for targeted transcripts in brain tissue (BA9; [10]and cerebral microvascular endothelial cells [11] from the same individuals used in this study (only those values with p<0.05 are shown). Columns 11 shows fold changes of the corresponding analytes in serum from 5 living cohorts of recent onset schizophrenia patients (n = 250) and control subjects (n = 230) [3]. Mean fold change over all significant cohorts is shown.

+ indicates an inconsistent fold change between centres. Column 10 indicates how many cohorts the analyte was significant in. Bold text indicates overlap between the brain analytes, mRNA transcripts or serum analytes. Reproducibility for serum analytes was only considered where significance occurred in at least 2 cohorts. TIMP-1 = tissue inhibitor of metalloproteinases-1, VCAM-1 = vascular cell adhesion molecule-1, PARC = pulmonary activation-regulated chemokine, VEGF = vascular endothelial growth factor, MIP-1β = macrophage inflammatory protein-1beta.

Samples from bipolar disorder patients were also analysed. Five analytes were significantly altered in bipolar disorder, however the false discovery rate was high in all cases (four out of five analytes had q values>0.9), both with and without the inclusion of Hb as a covariate (data not shown). This suggests that there is no strong biomarker profile for bipolar disorder in this tissue, considering the analytes targeted by the platform and given the differences in demographic variables between patients and controls. Similar results were obtained for bipolar disorder in frontal cortex of the same brain series by whole genome microarray [12].

### Validation

#### ELISA

**Figure 1:**
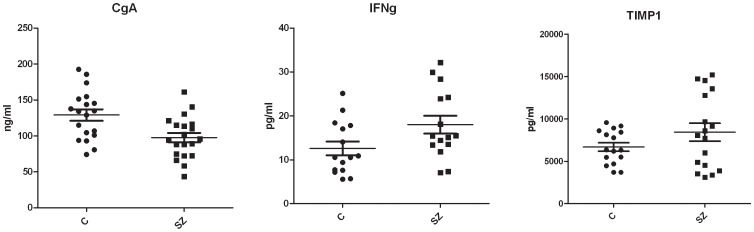
Enzyme-linked immunoadsorbent assay validation of changes in brain protein levels. A subset of the same brain samples used for multiplex immunoassay analysis was analyzed using uniplex ELISAs as indicated. All 3 proteins tested showed consistent results with the findings in the multiplex immunoassay analysis. Interferon-γ, *p* = 0.043, FC = 1.4; Chromogranin A, *p* = 0.004, FC = −1.3; tissue inhibitor of metalloproteinases 1 (TIMP-1), *p* = 0.15, FC 1.3. FC = fold change.

The results of three of the differentially expressed brain proteins were validated using uniplex ELISA tests in schizophrenia and control samples. This confirmed that interferon-γ (*p* = 0.043, FC = 1.4) and chromogranin A (*p* = 0.004, FC = −1.3) were present at significantly different levels between schizophrenia and control samples (Figure 1). The same analysis showed that TIMP-1 had a fold change consistent with the multiplex immunoassay data, although this did not reach significance (*p = *0.15, FC = 1.3). However, the uniplex ELISA and multiplex immunoassay results were significantly correlated for this analyte (*r = *0.87; *p*<0.0001).

#### mRNA levels

We have previously analyzed prefrontal cortex tissue from the same individuals at the mRNA transcript level using whole genome microarrays on both brain tissue homogenate and laser microdissected microvascular endothelium. As some of the analytes are expected to be localised to endothelium, we investigated mRNA levels from both datasets. In the case of the steroid hormone cortisol, transcripts for the glucocorticoid receptor (NR3C) were investigated. This showed that 9 molecules (NR3C receptor, TIMP1, VCAM1,, prostatic acid phosphatise, cancer antigen 125, chromogranin A, thrombopoeitin, immunoglobulin E, and MIP-1β) were significantly altered at both the protein and transcript levels, although fold changes were not generally in agreement (Table 3). Similar disagreements in direction of fold change between mRNA and the corresponding protein levels have been reported previously [13]. Interestingly, cortisol levels determined by multiplex immunoassay analysis were found to be negatively correlated with levels of the glucocorticoid receptor transcript (NR3C) in brain tissue homogenate from patients but not controls (*r* = −0.41; *p* = 0.017). This is consistent with downregulation of the glucocorticoid receptor by chronic hypercortisolemia. Conversely the glucocorticoid receptor appears to be upregulated in microvascular endothelium, providing further evidence for abnormalities in the cortisol pathway. This pathway has tissue-specific functions in the blood brain barrier, where glucocorticoid receptors are responsible for the closing of tight junctions [14].

### Univariate comparison of brain and serum analyte levels

The same molecules which were identified as significantly altered in *post mortem* brain tissues from schizophrenia patients were also analyzed in serum from living first onset schizophrenia (n = 250) and control (n = 230) subjects over 5 cohorts, using the same multiplex immunoassay platform. P-values were calculated in each cohort using two-tailed Wilcoxon rank-sum tests. This showed that 9 of the analytes (C-reactive protein, cortisol, resistin, TIMP-1, chromogranin A, VEGF, thrombopoetin, alpha-2 macroglobulin, and glutathione S-transferase) were also found to be reproducibly altered between schizophrenia and control subjects in serum (Table 3). In addition, 4 of these analytes showed opposite changes in brain and serum, which may indicate counter-regulation of these proteins between the brain and periphery.

### Multivariate comparison of brain and serum analyte levels

**Figure 2:**
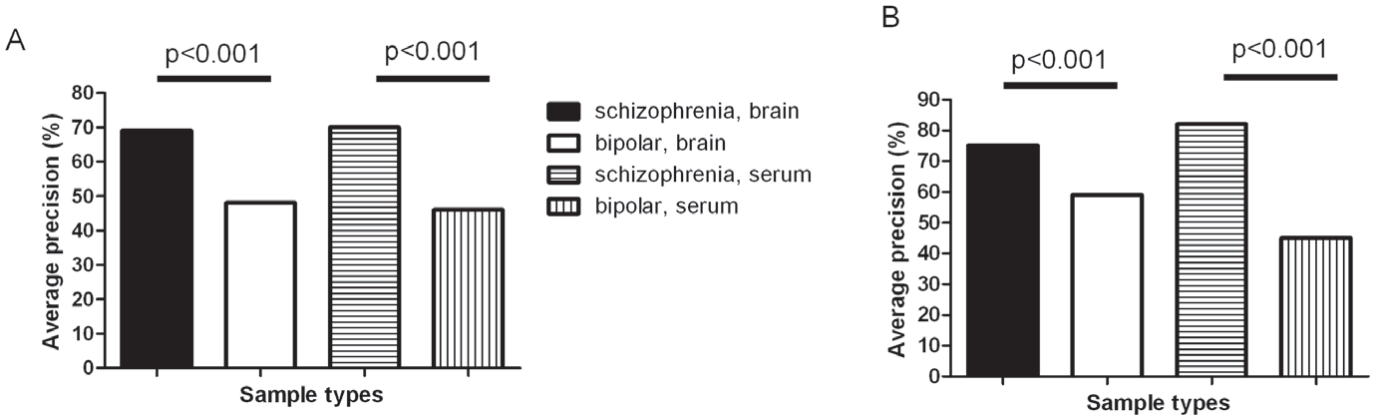
Brain vs serum analysis. Targeted analyte cluster analysis was performed to identify clusters of co-behaving analytes having the ability to distinguish schizophrenia from control with significantly greater precision than bipolar disorder from control. The cluster derived from brain tissue data showing the most similarity in serum data consisted of alpha-2 macroglobulin + plasminogen activator inhibitor-1 + prostatic acid phosphatase + serum glutamic oxaloacetic transaminase (A). The cluster derived from serum data showing the most similarity in brain data consisted of alpha-1 antitrypsin, alpha-2 macroglobulin + cortisol + sex hormone binding globulin + sortilin (B). (C) Ingenuity Pathways Analysis was performed on the cluster of co-behaving analytes derived from brain tissue (schizophrenia vs control) which gave most similar results in serum. The analysis shows that these four molecules (marked in green) interact via IL1β (marked in orange), which has previously been found altered in schizophrenia (see text for details). Solid lines represent physical interactions (i.e. binding), whereas arrows represent any other type of indirect cellular interactions. PAI-1 plasminogen activator inhibitor-1, GOT1 serum glutamic oxaloacetic transaminase, A2M alpha-1 macroglobulin, PAP prostatic acid phosphatase. Other members of network represented by HUGO gene symbols.

We used a targeted analyte cluster (TAC) approach to identify molecules which are correlated at the multivariate level in the brain dataset. This method identifies clusters of analytes which have the strongest influence on the data without necessarily requiring significance at the univariate level. These correlations are likely for molecules which are co-regulated as components of similar or identical molecular pathways. We investigated the brain data for clusters of analytes that could be used to distinguish schizophrenia from normal control subjects and then looked for those which gave high precision in both brain and serum, after correction for demographic variables. To further identify clusters that specifically give a good prediction power in distinguishing schizophrenia from controls but not bipolar disorder from controls, we also tested the ability of those schizophrenia-associated clusters to separate bipolar disorder patients from controls. As a result, thirteen clusters were identified in the brain dataset that distinguished between patients and controls with significantly higher precision in predicting schizophrenia than bipolar disorder; however, only four of these were also detectable in serum (Table S3a, Figure S1). A cluster consisting of alpha-2-macroglobulin, plasminogen activator inhibitor 1, prostatic acid phosphatase and serum glutamic oxaloacetic transaminase could distinguish schizophrenia from controls in both brain tissue and serum with a good precision (69% and 70%,respectively), but has a consistently low precision value in the case of distinguishing bipolar disorder from normal controls (<50% precision in both brain tissue and serum)(Figure 2A; Table S3b). An Ingenuity Pathway Analysis network indicates that these 4 molecules interact via IL1β, which shows a trend for differential expression in brain (p = 0.09) but not serum, and has previously been shown to be altered in schizophrenia CSF [15] and serum [16](Figure 2C). Therefore this cluster may provide an example of how altered brain biochemistry is reflected in serum analytical profile specifically for schizophrenia, and provides a useful starting point for further study. We performed the converse analysis by identifying clusters of analytes in serum which also gave high precision in brain tissue. 11 clusters were identified in the serum data that distinguished between patients and controls with significantly higher precision in schizophrenia than bipolar disorder (Table S4a, Figure S2). The cluster giving the best performance in both serum and brain tissue consisted of alpha-2 macroglobulin, cortisol, alpha-1 antitrypsin, sex hormone binding globulin and sortilin (Figure 2B, Table S4b).

## Discussion

Recent studies have shown that biomarkers for schizophrenia can be reliably detected in the bloodstream [3], [8]. However the relationship of these biomarkers to schizophrenia neuropathology is unclear. Here, we have shown that a subset of these analytes which are predominantly involved in the inflammatory response are differentially expressed in *post mortem* brain tissue from schizophrenia patients, and similar patterns of change can be observed in schizophrenia brain tissue as in the periphery. This provides further evidence for the systemic nature of schizophrenia and provides additional validity to the previously published peripheral biomarkers. However, there are a number of limitations of the study. Firstly the difference in brain regions (BA9/10) between microarray and multiplex immunoassay data limits the potential to observe comparable findings between the datasets. Secondly, the difference in patient selection between post mortem brain samples, generally obtained from patients who were drug treated either at time of death or during their life, and after several years of illness, and serum samples from living patients who were drug naive and suffering their first episode of schizophrenia, complicates comparison between sample sets. We have previously shown the serum biomarkers are robust before and after treatment and in chronic illness [8]. Nonetheless, a small number of analytes are correlated with either lifetime antipsychotic treatment or duration of illness as shown in Table S2, therefore these data should be interpreted with caution. Future studies should be carried out which link peripheral blood, brain imaging and post mortem studies within the same patients.

The co-ordination between the brain and periphery is likely to be mediated through the circulatory system which contains a number of secreted regulatory molecules and hormones which target multiple systems of the body, including the central nervous system. Hormones are produced in the diffuse neuroendocrine system which includes the hypothalamus, pituitary, adrenals, pancreatic islet cells, gonads, gut and adipose tissue. Previously, we have shown that the level of a number of hormones are altered in post mortem pituitary glands from schizophrenia patients compared to control subjects [17]. These included increased levels of pro-opiomelanocortin peptides and decreased levels of growth hormone, prolactin and arginine vasopressin. All of these molecules have been shown to have effects on various aspects of central nervous system function including inflammation [18], sleep [19], regulation of dopamine levels [20] and neurotrophic effects [21]. We have also demonstrated that the levels of hormones such as insulin, cortisol, progestone, prolactin, growth hormone and chromogranin A are altered in the circulation of first onset schizophrenia patients compared to controls [4].

Notably, insulin has been shown to cross the blood brain barrier and has a number of effects on central nervous system functions including alterations in ligand-gated ion channel trafficking and modulation of the tone of synaptic transmission by regulation of cell-surface expression of inhibitory and excitatory receptors [22]. Other studies have shown that insulin can have direct effects on synaptic plasticity and cognitive function [23]. In addition, emerging evidence suggests that central administration of insulin may enhance cognitive function in diseased and healthy individuals [24]. For example, insulin and insulin-like growth factor-1, or insulin-sensitizing agents), may offer novel disease-modifying neurocognitive treatments or protect against neurodegeration [25].

Although the multiplex immunoassay panel primarily targets blood analytes, the majority of the significant molecules identified here are known to be expressed in vascularised brain tissue. Furthermore, several of the significant analytes have been reported to be altered in previous studies of schizophrenia patients, although most of these changes have been identified in the periphery. C-reactive protein has been found at increased levels in serum from schizophrenia patients and this was associated with the severity of cognitive impairment [3], [8], [26], [27]. This protein is expressed in a variety of cell types including neurons [28]. Altered secretion of interferon-gamma from PBMCs has been reported in schizophrenia [29], [30], albeit with conflicting results, although altered brain expression has not previously been described. Immunoglobulin E has previously been shown to be increased in serum of treatment-resistant schizophrenia patients [31]; we found decreased levels of this protein in brain tissue. Immunoglobulin E receptors are expressed on peripheral neurons [32] but brain expression is unclear, despite the fact that other immunoglobulin subtypes are expressed in the brain [33]. There is some evidence for altered expression of plasminogen activator inhibitor-1 in drug-treated schizophrenia patients [34]. Decreased levels of vascular endothelial growth factor have previously been reported in the dorsolateral prefrontal cortex of schizophrenia patients [35], consistent with the findings of our study. A further link with published data on schizophrenia is provided by prostatic acid phosphatase, seen to be increased here, which dephosphorylates Erbb2, a receptor for the schizophrenia susceptibility gene product neuregulin [36].

For some of the analytes that we have identified here, there is no reported expression in neuronal, glial or vascular cell types (cancer antigen 19-9, cancer antigen 125, MIP1-beta, cortisol). This suggests that the presence of such analytes in the brain could be due to blood contamination. However, we have excluded the possibility that the differences in the levels of these molecules were due to inter-sample variability in blood contamination. In the case of cortisol, its presence in the brain may be through binding or internalization in target tissues. The finding of increased levels of cortisol in schizophrenia patients is well-documented. However, the function and/or location of other molecules in the brain is not clear (cancer antigen 19-9, cancer antigen 125, MIP1-beta) and further work is required in these cases.

The finding of alterations in the levels of several endothelial-related markers is of interest and adds to previous evidence for abnormal blood-brain barrier function in schizophrenia [11]. For example, we found increased levels of adiponectin in the brain and this protein is known to accumulate in damaged blood vessels. Furthermore the matrix metalloproteinase 1/tissue inhibitor of metalloproteinase 1 (MMP1/TIMP1) pathway may contribute to the breakdown of the blood brain barrier in conditions such as Alzheimer's disease [37] and diabetes [38]. Our finding of increased TIMP1 and decreased MMP1 is consistent with previous reports and suggests a cytoprotective role of this pathway in the brains of schizophrenia patients. The proteins vascular cell adhesion molecule (VCAM1) and vascular endothelial growth factor (VEGF) showed opposite directions of change in brain and serum. VEGF has previously been reported to be decreased in schizophrenia brain [35], consistent with the present findings. Circulating levels of this molecule are increased following hypoglycaemia and this effect is associated with preserved cognitive function under hypoglycaemic conditions [39]. Thus the dysregulation of VEGF seen here may reflect an attempt to counteract abnormal glucose handling in the schizophrenia brain. VCAM1 is expressed on cytokine-activated endothelium. Therefore, the altered levels of this protein found in this study are consistent with an abnormal inflammatory status in the cerebral microvasculature of schizophrenia patients [11]. The function of the circulating form is unclear but is thought to be associated with microvascular dysfunction [40] and/or atherosclerotic damage [41].

### Conclusions

Here we found changes in analytes in *post mortem* brains of schizophrenia patients which appear to be linked to changes in the same molecules in circulation of living schizophrenia patients. We propose a model in which the central nervous system and periphery are linked via components of the diffuse neuroendocrine system, such as cortisol, insulin, leptin, pro-opiomelanocortin, prolactin and growth hormone. However, it should be noted that to fully investigate the systemic nature of schizophrenia, prospective studies must be carried out which link peripheral blood, brain imaging and post mortem studies within the same patients. Further studies in this area are important to increase our understanding of the pathophysiological pathways underlying schizophrenia and other psychiatric disorders which, in turn, could lead to novel beneficial strategies for therapeutic intervention.

## Supporting Information

Figure S1 **Graphical representation of the results of the brain-to-serum TAC analysis.** Word document.
(DOCX)

Figure S2 **Graphical representation of the results of the serum-to-brain TAC analysis.** Word document.
(DOCX)

Table S1 The complete list of analytes investigated on the MyriadRBM DiscoveryMAP platform July 2009. Excel file.
(XLSX)

Table S2 **Extended results of the ANCOVA analysis.** Excel file.
(XLSX)

Table S3 **Additional information on the brain-to-serum TAC analysis.** A: Extended results of the brain-to-serum TAC analysis. Word document. B: Fold changes of analytes included in the results of the brain-to-serum TAC analysis. Word document.
(DOCX)

Table S4 **Additional information on the serum-to-brain TAC analysis.** A: Extended results of the serum-to-brain TAC analysis. Word document. B: Fold changes of analytes included in the results of the serum-to-brain TAC analysis. Word document.
(DOCX)
