# Supplementary material for: Comparison of Peripheral and Central Schizophrenia Biomarker Profiles
Source: PLoS One. 2012 Oct 30;7(10):e46368. doi: 10.1371/journal.pone.0046368 (PMC3484150; doi:10.1371/journal.pone.0046368)
Supplement: Figure S1 — Graphical representation of the results of the brain-to-serum TAC analysis. Word document. (DOCX) [file pone.0046368.s001.docx]

**Fig S1.**

Graphical representation of the precision of top clusters in distinguishing disease from control (see Table S3a for further details). All three clusters showed comparable precision in distinguishing schizophrenia from control in serum as well as brain, and could distinguish schizophrenia from control with significantly greater precision than bipolar from control. ACE angiotensin converting enzyme, ACTH adrenocorticotrophic hormone, NGFb nerve growth factor beta, PARC Pulmonary and Activation-Regulated Chemokine, TNF-beta tumour necrosis factor beta, IFN-gamma interferon gamma, PAI-1 plasminogen activator inhibitor, SGOT Serum Glutamic Oxaloacetic Transaminase.
