# Supplementary material for: Comparison of Peripheral and Central Schizophrenia Biomarker Profiles
Source: PLoS One. 2012 Oct 30;7(10):e46368. doi: 10.1371/journal.pone.0046368 (PMC3484150; doi:10.1371/journal.pone.0046368)
Supplement: Figure S2 — Graphical representation of the results of the serum-to-brain TAC analysis. Word document. (DOCX) [file pone.0046368.s002.docx]

**Fig S2**

Graphical representation of the precision of eleven clusters in distinguishing disease from control in the serum data (see Table S4a for further details).The majority of these clusters did not show equivalent discriminatory power in the brain data. However, a cluster consisting of cortisol, alpha-1-antitrypsin, alpha-2-macroglobulin, sex hormone binding globulin and sortilin did show equivalent precision in both datasets. A1A alpha-1 antitrypsin, A2M alpha-2 macroglobulin, BDNF brain derived neurotrophic factor, CRP C reactive protein, FSH follicle stimulating hormone, LH luteinizing hormone, SHBG sex hormone binding globulin, TNFRII tumor necrosis factor 2, VEGF Vascular endothelial growth factor.
