# Supplementary material for: Comparison of Peripheral and Central Schizophrenia Biomarker Profiles
Source: PLoS One. 2012 Oct 30;7(10):e46368. doi: 10.1371/journal.pone.0046368 (PMC3484150; doi:10.1371/journal.pone.0046368)
Supplement: Table S3 — Additional information on the brain-to-serum TAC analysis. A: Extended results of the brain-to-serum TAC analysis. Word document. B: Fold changes of analytes included in the results of the brain-to-serum TAC analysis. Word document. (DOCX) [file pone.0046368.s005.docx]

| Protein types | Analyte clusters | Average precision (%) | |
| --- | --- | --- | --- |
|  |  | SZ vs. NC | BD vs. NC |
| Metabolic | adiponectin + glucagon + insulin + resistin | 63 | 47 |
|  | ACE + ACTH + angiotensinogen + prolactin | 63 | 50 |
| Immune | IFN-gamma + IL-6 receptor + NGFb + PARC + TNF-beta | 79 | 38 |
|  | CRP + IFN-gamma + NGFb + PARC + TNF-beta | 78 | 39 |
|  | IFN-gamma + M-CSF + NGFb + PARC + TNF-beta | 78 | 41 |
| Metabolic & Immune | ACE + insulin + IFN-gamma + PARC + TNF-beta | 83 | 45 |
|  | glucagon + insulin + IFN-gamma + PARC + TNF-beta | 82 | 45 |
|  | insulin + IFN-gamma + PARC + TNF-beta | 80 | 47 |
| other | *Alpha-2 Macroglobulin + PAI-1 + Prostatic Acid Phosphatase + SGOT | 69 | 48 |
|  | *PAI-1 + Prostatic Acid Phosphatase + SGOT | 68 | 47 |
|  | *Alpha-2 Macroglobulin + PAI-1 | 61 | 46 |
| All | ACE + IFN-gamma + PARC + TNF-beta + PAI-1 | 80 | 42 |
|  | resistin +IFN-gamma +TNF-beta + PAI-1 + prostatic acid phosphatase | 75 | 64 |

**Table S3a & b.** Additional information on the brain-to-serum TAC analysis

**Table S3a**. Thirteen clusters of analytes identified in MAP data from brain tissue having the ability to distinguish schizophrenia from control with significantly greater precision than bipolar disorder from control. Of these clusters, only three exclusively contained analytes which could be measured in serum (marked with an asterisk). These brain clusters were then tested to assess their discriminatory power in the serum data. All three clusters showed comparable precision in distinguishing schizophrenia from control in serum as well as brain (see fig S1). ACE angiotensin converting enzyme, ACTH adrenocorticotrophic hormone, NGFb nerve growth factor beta, PARC Pulmonary and Activation-Regulated Chemokine, TNF-beta tumour necrosis factor beta, IFN-gamma interferon gamma, PAI-1 plasminogen activator inhibitor, SGOT Serum Glutamic Oxaloacetic Transaminase.

**Table S3b. Fold changes for the individual analytes in the top cluster (see main text for details).** TAC analysis is a powerful method to identify small groups of co-behaving analytes which may not be easily identified using univariate statistics. Here we show the individual fold changes for analytes in the top cluster. The TAC results show that these molecules co-behave in schizophrenia brain tissue, and also in serum, but not in bipolar disorder. However the individual fold changes may be insignificant or dissimilar between datasets. This highlights the power of the TAC method to identify similarities between datasets which are not visible by examining individual fold changes at a univariate level.

| **Analyte** | **FC brain SZ/con** | **FC brain BD/con** | **FC serum SZ/con** | **FC serum BD/con** |
| --- | --- | --- | --- | --- |
| Alpha-2 macroglobulin | -1.1 | 1.0 | 1.2 | -1.3 |
| PAI-1 | 1.6 | 1.2 | 1.0 | -1.1 |
| Prostatic acid phosphatase | 1.1 | 1.0 | -1.1 | 1.0 |
| SGOT | 1.0 | 1.1 | 1.2 | 1.1 |

(fold change values are unadjusted for demographic variables)
