# Supplementary material for: Comparison of Peripheral and Central Schizophrenia Biomarker Profiles
Source: PLoS One. 2012 Oct 30;7(10):e46368. doi: 10.1371/journal.pone.0046368 (PMC3484150; doi:10.1371/journal.pone.0046368)
Supplement: Table S4 — Additional information on the serum-to-brain TAC analysis. A: Extended results of the serum-to-brain TAC analysis. Word document. B: Fold changes of analytes included in the results of the serum-to-brain TAC analysis. Word document. (DOCX) [file pone.0046368.s006.docx]

**Tables S4 a & b.** Additional information on the serum-to-brain TAC analysis.

**Table S4a.** The same analysis as described above was performed in the serum data (centre 1 only). Eleven clusters were identified which have the ability to distinguish schizophrenia from control with significantly greater precision than bipolar disorder from control. Clusters containing testosterone were removed since this analyte was highly associated with gender. These serum clusters were then tested to assess their discriminatory power in the brain data (fig S2). The majority of these clusters did not show equivalent discriminatory power in the brain data. However, a cluster consisting of cortisol, alpha-1-antitrypsin, alpha-2-macroglobulin, sex hormone binding globulin and sortilin did show equivalent precision in both datasets. A1A alpha-1 antitrypsin, A2M alpha-2 macroglobulin, BDNF brain derived neurotrophic factor, CRP C reactive protein, FSH follicle stimulating hormone, LH luteinizing hormone, SHBG sex hormone binding globulin, TNFRII tumor necrosis factor 2, VEGF Vascular endothelial growth factor.

| Protein types | Analyte clusters | Average precision (%) | |
| --- | --- | --- | --- |
|  |  | SZ vs. NC | BD vs. NC |
| Metabolic | Cortisol + FSH + LH | 64 | 46 |
| Immune | CD40 + IL-16 + myeloperoxidase | 71 | 49 |
|  | Complement 3 + CRP + IL-16 + TNF RII | 57 | 51 |
|  | CD40 + myeloperoxidase | 65 | 51 |
| Metabolic & Immune | LH + CD40 + complement 3 + IL_16 + TNF-RII | 60 | 54 |
|  | LH + complement 3 + IL_16 | 60 | 51 |
| other | Alpha-1 antitrypsin + alpha-2 macroglobulin + SHBG + sortilin | 80 | 47 |
|  | Alpha-1 antitrypsin + alpha-2 macroglobulin + sortilin + thyroxine binding globulin | 77 | 50 |
|  | Alpha-1 antitrypsin + alpha-2 macroglobulin + BDNF + SHBG + VEGF | 73 | 43 |
| All | Cortisol + alpha-1 antitrypsin + alpha-2 macroglobulin + SHBG + sortilin | 81 | 45 |
|  | Cortisol + alpha-2 macroglobulin + SHBG + sortilin + VEGF | 78 | 43 |

**Table S4b. Fold changes for the individual analytes in the top cluster (see main text for details).**TAC analysis is a powerful method to identify small groups of co-behaving analytes which may not be easily identified using univariate statistics. Here we show the individual fold changes for analytes in the top cluster. The TAC results show that these molecules co-behave in schizophrenia serum, and also brain tissue, but not in bipolar disorder. However the individual fold changes may be insignificant or dissimilar between datasets as shown below. This highlights the power of the TAC method to identify similarities between datasets which are not visible by examining individual fold changes at a univariate level.

| **Analyte** | **FC brain SZ/con** | **FC brain BD/con** | **FC serum SZ/con** | **FC serum BD/con** |
| --- | --- | --- | --- | --- |
| Alpha-1 antitrypsin | 1.3 | 1.2 | 1.1 | -1.0 |
| Alpha-2 macroglobulin | -1.1 | 1.0 | 1.2 | -1.3 |
| Cortisol | 1.8 | 1.4 | 1.1 | 1.1 |
| SHBG | -1.1 | 2.0 | -1.4 | -1.8 |
| sortilin | -1.1 | 1.0 | -1.3 | 1.1 |

(fold change values are unadjusted for demographic variables)
